# Supplementary material for: Cardio-metabolic traits and its socioeconomic differentials among school children including metabolically obese normal weight phenotypes in India: A post-COVID baseline characteristics of LEAP-C cohort
Source: PLoS One. 2025 May 6;20(5):e0321898. doi: 10.1371/journal.pone.0321898 (PMC12054912; doi:10.1371/journal.pone.0321898)
Supplement: Supporting Information — (DOCX) [file pone.0321898.s001.docx]

| Characteristics | *Children (6-9 Years)* | | | | *Adolescents (10-19 Years)* | | | | *Overall (6-19 Years)* | | | |
| --- | --- | --- | --- | --- | --- | --- | --- | --- | --- | --- | --- | --- |
|  | ***Total***  *Boys: n=657*  *Girls: n=570* | ***Public***  *Boys: n=276*  *Girls: n=227* | ***Private***  *Boys: n=381*  *Girls: n=343* | ***p-value*** | ***Total***  *Boys: n=1558*  *Girls: n=1071* | ***Public***  *Boys: n=876*  *Girls: n=587* | ***Private***  *Boys: n=682*  *Girls: n=484* | ***p-value*** | ***Total***  *Boys: n=2215*  *Girls: n=1641* | ***Public***  *Boys: n=1152*  *Girls: n=814* | ***Private***  *Boys: n=1063*  *Girls: n=827* | ***p-value*** |
|  | **mean±SD** | **mean±SD** | **mean±SD** |  | **mean±SD** | **mean±SD** | **mean±SD** |  | **mean±SD** | **mean±SD** | **mean±SD** |  |
| *Height (cm)* |  |  |  |  |  |  |  |  |  |  |  |  |
| Total | 128.05±8.78 | 124.03±7.94 | 130.84±8.25 | <0.001 | 152.05±11.55 | 150.03±11.44 | 154.59±11.19 | <0.001 | 144.41±15.51 | 143.38±15.57 | 145.49±15.38 | <0.001 |
| Boys | 128.57±8.53 | 124.89±7.61 | 131.24±8.17 | <0.001 | 154.14±12.58 | 152.48±12.49 | 156.27±12.40 | <0.001 | 146.56±16.41 | 145.87±16.47 | 147.30±16.33 | 0.0410 |
| Girls | 127.44±9.03 | 122.98±8.21 | 130.39±8.32 | <0.001 | 149.02±9.04 | 146.38±8.46 | 152.22±8.69 | <0.001 | 141.52±13.68 | 139.86±13.44 | 143.16±13.74 | <0.001 |
| p-value | 0.024 | 0.007 | 0.164 |  | <0.001 | <0.001 | <0.001 |  | <0.001 | <0.001 | <0.001 |  |
| *Weight (kg)* |  |  |  |  |  |  |  |  |  |  |  |  |
| Total | 27.39±8.42 | 23.12±5.28 | 30.35±8.91 | <0.001 | 44.26±13.45 | 39.89±11.11 | 49.75±14.11 | <0.001 | 38.89±14.41 | 35.60±12.35 | 42.32±15.56 | <0.001 |
| Boys | 27.85±8.60 | 23.63±5.35 | 30.90±9.21 | <0.001 | 45.32±14.34 | 40.96±11.95 | 50.92±15.19 | <0.001 | 40.14±15.17 | 36.81±13.04 | 43.74±16.45 | <0.001 |
| Girls | 26.85±8.17 | 22.51±5.14 | 29.73±8.54 | <0.001 | 42.73±11.88 | 38.29±9.51 | 48.11±12.24 | <0.001 | 37.21±13.13 | 33.89±11.07 | 40.49±14.14 | <0.001 |
| P-Value | 0.039 | 0.018 | 0.077 |  | <0.001 | <0.001 | <0.001 |  | <0.001 | <0.001 | <0.001 |  |
| *BMI^$^ (kg/m^2^)* |  |  |  |  |  |  |  |  |  |  |  |  |
| Total | 16.39±3.24 | 14.88±2.11 | 17.44±3.47 | <0.001 | 18.82±4.06 | 17.46±3.34 | 20.54±4.22 | <0.001 | 18.05±3.98 | 16.80±3.27 | 19.35±4.23 | <0.001 |
| Boys | 16.53±3.31 | 15.00±2.15 | 17.64±3.56 | <0.001 | 18.71±4.09 | 17.30±3.30 | 20.53±4.30 | <0.001 | 18.07±4.00 | 16.75±3.22 | 19.49±4.27 | <0.001 |
| Girls | 16.22±3.15 | 14.73±2.04 | 17.21±3.36 | <0.001 | 18.98±3.99 | 17.69±3.37 | 20.56±4.12 | <0.001 | 18.03±3.95 | 16.86±3.33 | 19.17±4.16 | <0.001 |
| P-Value | 0.096 | 0.149 | 0.097 |  | 0.092 | 0.031 | 0.898 |  | 0.751 | 0.459 | 0.101 |  |
| *WC^@^ (cm)* |  |  |  |  |  |  |  |  |  |  |  |  |
| Total | 56.15±8.97 | 52.44±6.11 | 58.72±9.71 | <0.001 | 65.70±11.11 | 61.69±9.01 | 70.74±11.43 | <0.001 | 62.66±11.38 | 59.32±9.29 | 66.13±12.28 | <0.001 |
| Boys | 56.85±9.27 | 53.69±6.53 | 59.14±10.25 | <0.001 | 67.63±11.41 | 63.63±9.22 | 72.77±11.90 | <0.001 | 64.43±11.89 | 61.25±9.63 | 67.89±13.08 | <0.001 |
| Girls | 55.34±8.55 | 50.91±5.19 | 58.26±9.07 | <0.001 | 62.89±10.01 | 58.79±7.85 | 67.86±10.08 | <0.001 | 60.27±10.18 | 56.60±8.03 | 63.88±10.77 | <0.001 |
| P-Value | 0.003 | <0.001 | 0.227 |  | <0.001 | <0.001 | <0.001 |  | <0.001 | <0.001 | <0.001 |  |
| *HC^#^ (cm)* |  |  |  |  |  |  |  |  |  |  |  |  |
| Total | 65.47±8.73 | 60.46±6.11 | 68.95±8.58 | <0.001 | 79.28±10.55 | 75.46±9.16 | 84.07±10.23 | <0.001 | 74.89±11.89 | 71.62±10.71 | 78.28±12.11 | <0.001 |
| Boys | 65.04±8.83 | 60.17±6.22 | 68.57±8.77 | <0.001 | 78.59±10.73 | 74.90±9.35 | 83.33±10.52 | <0.001 | 74.57±11.93 | 71.37±10.74 | 78.04±12.19 | <0.001 |
| Girls | 65.97±8.58 | 60.82±5.97 | 69.38±8.35 | <0.001 | 80.28±10.21 | 76.30±8.79 | 85.11±9.73 | <0.001 | 75.31±11.83 | 71.98±10.67 | 78.58±12.01 | <0.001 |
| P-Value | 0.064 | 0.239 | 0.206 |  | <0.001 | 0.004 | 0.003 |  | 0.056 | 0.212 | 0.332 |  |
| *MUAC^*^ (cm)* |  |  |  |  |  |  |  |  |  |  |  |  |
| Total | 18.74±3.19 | 17.06±2.09 | 19.91±3.30 | <0.001 | 22.38±3.95 | 20.83±3.36 | 24.32±3.77 | <0.001 | 21.22±4.09 | 19.87±3.50 | 22.63±4.19 | <0.001 |
| Boys | 18.69±3.25 | 17.13±2.08 | 19.81±3.48 | <0.001 | 22.62±4.08 | 21.10±3.56 | 24.58±3.86 | <0.001 | 21.45±4.25 | 20.15±3.68 | 22.87±4.38 | <0.001 |
| Girls | 18.80±3.12 | 16.97±2.11 | 20.01±3.09 | <0.001 | 22.03±3.72 | 20.44±2.98 | 23.96±3.62 | <0.001 | 20.91±3.84 | 19.48±3.17 | 22.32±3.92 | <0.001 |
| P-Value | 0.537 | 0.394 | 0.425 |  | <0.001 | <0.001 | 0.006 |  | <0.001 | <0.001 | 0.005 |  |

*^$^Body Mass Index, ^@^Waist Circumference, ^#^Hip Circumference, ^*^Mid-Upper Arm Circumference*

**S1 Table:** Anthropometric profile by gender and school type in children and adolescents (n=3856).

| *Clinical profile* | *Adolescents (10-19 Years)* | | | |
| --- | --- | --- | --- | --- |
|  | ***Total***  *Boys: n=1355*  *Girls: n=926* | ***Public***  *Boys: n=783*  *Girls: n=521* | ***Private***  *Boys: n=572*  *Girls: n=405* | ***p-value*** |
|  | **mean±SD** | **mean±SD** | **mean±SD** |  |
| *Systolic blood pressure (mmHg)* |  |  |  |  |
| Total | 105.39±9.99 | 104.18±10.06 | 106.99±9.67 | <0.001 |
| Boys | 106.11±10.38 | 104.99±10.48 | 107.64±10.06 | <0.001 |
| Girls | 104.33±9.28 | 102.97±9.27 | 106.08±9.02 | <0.001 |
| p-value | <0.001 | <0.001 | 0.013 |  |
| *Diastolic blood pressure (mmHg)* |  |  |  |  |
| Total | 67.91±7.13 | 67.38±7.29 | 68.62±6.83 | <0.001 |
| Boys | 67.40±7.18 | 66.99±7.19 | 67.95±7.13 | 0.015 |
| Girls | 68.66±6.99 | 67.96±7.42 | 69.56±6.28 | <0.001 |
| p-value | <0.001 | 0.018 | <0.001 |  |
| *Metabolic profile* | *Boys: n=1300*  *Girls: n=897* | *Boys: n=771*  *Girls: n=510* | *Boys: n=529*  *Girls: n=387* | ***p-value*** |
| *Fasting plasma glucose (mg/dl)* |  |  |  |  |
| Total | 92.60±8.65 | 90.43±8.17 | 95.64±8.38 | <0.001 |
| Boys | 93.03±8.76 | 90.84±8.47 | 96.22±8.20 | <0.001 |
| Girls | 91.98±8.44 | 89.81±7.66 | 94.85±8.58 | <0.001 |
| p-value | 0.005 | 0.027 | 0.015 |  |
| *Fasting insulin (µIU/ml)* |  |  |  |  |
| Total | 10.92±8.01 | 9.59±7.61 | 12.77±8.18 | <0.001 |
| Boys | 10.29±8.17 | 9.08±8.40 | 12.04±7.49 | <0.001 |
| Girls | 11.84±7.68 | 10.37±6.17 | 13.77±8.95 | <0.001 |
| p-value | <0.001 | 0.003 | 0.001 |  |
| *HOMA-IR^#^* |  |  |  |  |
| Total | 2.54±2.23 | 2.14±1.50 | 3.10±2.87 | <0.001 |
| Boys | 2.39±1.95 | 2.03±1.54 | 2.92±2.33 | <0.001 |
| Girls | 2.75±2.56 | 2.31±1.42 | 3.33±3.46 | <0.001 |
| p-value | <0.001 | <0.001 | 0.032 |  |
| *Total cholesterol (mg/dl)* |  |  |  |  |
| Total | 146.42±28.98 | 141.52±28.55 | 153.27±28.18 | <0.001 |
| Boys | 143.39±29.27 | 137.18±27.89 | 152.43±28.89 | <0.001 |
| Girls | 150.81±27.98 | 148.07±28.31 | 154.41±27.16 | <0.001 |
| p-value | <0.001 | <0.001 | 0.295 |  |
| *Triglyceride (mg/dl)* |  |  |  |  |
| Total | 82.47±33.54 | 81.00±33.16 | 84.53±33.96 | 0.015 |
| Boys | 80.51±34.01 | 77.60±32.16 | 84.75±36.22 | <0.001 |
| Girls | 85.31±32.64 | 86.12±34.08 | 84.23±30.65 | 0.389 |
| p-value | 0.001 | <0.001 | 0.819 |  |
| *Low-density lipoprotein (mg/dl)* |  |  |  |  |
| Total | 82.58±24.49 | 78.80±24.82 | 87.87±23.02 | <0.001 |
| Boys | 80.19±24.91 | 75.20±24.57 | 87.47±23.60 | <0.001 |
| Girls | 86.04±23.46 | 84.24±24.22 | 88.41±22.21 | 0.008 |
| p-value | <0.001 | <0.001 | 0.545 |  |
| *High-density lipoprotein (mg/dl)* |  |  |  |  |
| Total | 47.34±10.35 | 46.52±10.03 | 48.50±10.69 | <0.001 |
| Boys | 47.09±10.45 | 46.46±10.24 | 48.01±10.69 | 0.008 |
| Girls | 47.71±10.21 | 46.61±9.71 | 49.16±10.67 | <0.001 |
| p-value | 0.168 | 0.792 | 0.108 |  |

***^#^****Homeostatic Model Assessment for Insulin Resistance, HOMA-IR=((Fasting insulin(µIU/ml)*Fasting plasma glucose(mg/dl)/(22.5*18.0156))*

**S2 Table:** Clinical (n=2281) and metabolic (n=2197) profile by gender and school type in adolescents.

| Characteristics | Prevalence (95% CI) by WHO^#^ Criteria [in %] | | | Prevalence (95% CI) by IOTF^@^ Criteria [in %] | | | Prevalence (95% CI) by IAP^$^ Criteria [in %] | | |
| --- | --- | --- | --- | --- | --- | --- | --- | --- | --- |
|  | **Total** | **Boys** | **Girls** | **Total** | **Boys** | **Girls** | **Total** | **Boys** | **Girls** |
| *Children (6−9 years)* |  |  |  |  |  |  |  |  |  |
| Underweight |  |  |  |  |  |  |  |  |  |
| Total | 8.64 (7.13−10.35) | 7.46 (5.57−9.74) | 10.00 (7.66−12.76) | 25.59 (23.17−28.13) | 24.81 (21.55−28.30) | 26.49 (22.91−30.32) | 4.07 (3.04−5.34) | 3.35 (2.11−5.03) | 4.91 (3.29−7.02) |
| Public | 15.51 (12.45−18.97) | 13.41 (9.62−18.00) | 18.06 (13.28−23.69) | 40.56 (36.23−44.99) | 38.41 (32.64−44.42) | 43.17 (36.64−49.89) | 7.75 (5.57−10.45) | 6.52 (3.91−10.11) | 9.25 (5.82−13.79) |
| Private | 3.87 (2.58−5.54) | 3.15 (1.64−5.44) | 4.66 (2.69−7.46) | 15.19 (12.66−18.02) | 14.96 (11.53−18.94) | 15.45 (11.79−19.72) | 1.52 (0.76−2.70) | 1.05 (0.29−2.67) | 2.04 (0.82−4.16) |
| Overweight |  |  |  |  |  |  |  |  |  |
| Total | 12.63 (10.82−14.62) | 10.50 (8.26−13.10) | 15.09 (12.25−18.29) | 13.94 (12.05−16.00) | 12.02 (9.64−14.76) | 16.14 (13.21−19.42) | 15.00 (13.04−17.12) | 14.46 (11.86−17.38) | 15.61 (12.73−18.86) |
| Public | 3.78 (2.29−5.84) | 2.90 (1.26−5.63) | 4.85 (2.44−8.50) | 4.17 (2.60−6.31) | 3.62 (1.75−6.56) | 4.85 (2.44−8.50) | 6.36 (4.39−8.86) | 6.52 (3.91−10.11) | 6.17 (3.41−10.13) |
| Private | 18.78 (16.00−21.82) | 16.01 (12.47−20.09) | 21.87 (17.61−26.62) | 20.72 (17.82−23.86) | 18.11 (14.37−22.35) | 23.62 (19.22−28.47) | 20.99 (18.08−24.15) | 20.21 (16.29−24.60) | 21.87 (17.61−26.62) |
| Obesity |  |  |  |  |  |  |  |  |  |
| Total | 12.55 (10.75−14.54) | 15.53 (12.84−18.52) | 9.12 (6.89−11.79) | 7.58 (6.16−9.21) | 8.83 (6.77−11.26) | 6.14 (4.31−8.44) | 15.89 (13.89−18.06) | 18.87 (15.95−22.08) | 12.46 (9.86−15.45) |
| Public | 2.58 (1.38−4.38) | 3.26 (1.50−6.10) | 1.76 (0.48−4.45) | 1.39 (0.56−2.85) | 1.81 (0.59−4.18) | 0.88 (0.11−3.15) | 3.98 (2.45−6.07) | 4.71 (2.53−7.92) | 3.08 (1.25−6.25) |
| Private | 19.48 (16.65−22.55) | 24.41 (20.18−29.04) | 13.99 (10.5−18.12) | 11.88 (9.61−14.46) | 13.91 (10.60−17.80) | 9.62 (6.72−13.25) | 24.17 (21.09−27.46) | 29.13 (24.62−33.98) | 18.66 (14.68−23.19) |
| Overweight/Obesity |  |  |  |  |  |  |  |  |  |
| Total | 25.18 (22.78−27.71) | 26.03 (22.71−29.56) | 24.21 (20.75−27.94) | 21.52 (19.25−23.92) | 20.85 (17.81−24.16) | 22.28 (18.93−25.92) | 30.89 (28.31−33.56) | 33.33 (29.73−37.08) | 28.07 (24.42−31.95) |
| Public | 6.36 (4.39−8.86) | 6.16 (3.63−9.68) | 6.61 (3.75−10.66) | 5.57 (3.73−7.95) | 5.43 (3.07−8.81) | 5.73 (3.08−9.59) | 10.34 (7.82−13.34) | 11.23 (7.76−15.56) | 9.25 (5.82−13.79) |
| Private | 38.26 (34.70−41.91) | 40.42 (35.45−45.54) | 35.86 (30.78−41.19) | 32.60 (29.19−36.15) | 32.02 (27.36−36.96) | 33.24 (28.27−38.50) | 45.17 (41.50−48.87) | 49.34 (44.21−54.48) | 40.52 (35.29−45.93) |
| *Early Adolescents (10−14 years)* |  |  |  |  |  |  |  |  |  |
| Underweight |  |  |  |  |  |  |  |  |  |
| Total | 13.22 (11.81−14.73) | 15.84 (13.86−17.98) | 9.54 (7.69−11.66) | 25.36 (23.53−27.26) | 26.64 (24.21−29.18) | 23.57 (20.82−26.50) | 5.32 (4.41−6.36) | 5.52 (4.32−6.93) | 5.05 (3.71−6.70) |
| Public | 20.09 (17.82−22.51) | 23.61 (20.47−26.98) | 15.06 (11.98−18.59) | 37.24 (34.45−40.10) | 39.44 (35.75−43.22) | 34.10 (29.86−38.54) | 8.19 (6.68−9.92) | 8.50 (6.52−10.86) | 7.74 (5.51−10.51) |
| Private | 5.10 (3.81−6.66) | 6.51 (4.63−8.87) | 3.15 (1.69−5.32) | 11.31 (9.40−13.47) | 11.27 (8.79−14.16) | 11.38 (8.48−14.84) | 1.94 (1.17−3.01) | 1.94 (0.97−3.44) | 1.94 (0.84−3.78) |
| Overweight |  |  |  |  |  |  |  |  |  |
| Total | 15.83 (14.31−17.45) | 16.64 (14.62−18.82) | 14.70 (12.44−17.20) | 15.60 (14.09−17.21) | 16.16 (14.16−18.32) | 14.81 (12.55−17.32) | 16.21 (14.67−17.84) | 15.84 (13.86−17.98) | 16.72 (14.33−19.34) |
| Public | 6.55 (5.20−8.13) | 7.18 (5.36−9.39) | 5.65 (3.76−8.11) | 6.12 (4.81−7.66) | 6.45 (4.73−8.56) | 5.65 (3.76−8.11) | 8.10 (6.60−9.83) | 7.33 (5.49−9.55) | 9.21 (6.77−12.16) |
| Private | 26.81 (24.06−29.70) | 27.99 (24.34−31.88) | 25.18 (21.07−29.66) | 26.81 (24.06−29.70) | 27.82 (24.17−31.70) | 25.42 (21.29−29.91) | 25.79 (23.08−28.65) | 26.06 (22.49−29.87) | 25.42 (21.29−29.91) |
| Obesity |  |  |  |  |  |  |  |  |  |
| Total | 8.83 (7.66−10.11) | 10.40 (8.76−12.23) | 6.62 (5.08−8.46) | 5.18 (4.28−6.21) | 6.00 (4.75−7.46) | 4.04 (2.85−5.55) | 13.26 (11.86−14.78) | 15.68 (13.71−17.82) | 9.88 (8.00−12.03) |
| Public | 3.10 (2.18−4.27) | 3.67 (2.39−5.36) | 2.30 (1.15−4.08) | 1.81 (1.12−2.75) | 2.20 (1.24−3.60) | 1.26 (0.46−2.71) | 4.66 (3.52−6.03) | 5.72 (4.10−7.73) | 3.14 (1.77−5.12) |
| Private | 15.60 (13.38−18.02) | 18.49 (15.38−21.93) | 11.62 (8.69−15.11) | 9.17 (7.44−11.16) | 10.56 (8.16−13.39) | 7.26 (4.95−10.21) | 23.45 (20.83−26.23) | 27.64 (24.00−31.52) | 17.68 (14.12−21.70) |
| Overweight/Obesity |  |  |  |  |  |  |  |  |  |
| Total | 24.66 (22.85−26.54) | 27.04 (24.59−29.59) | 21.32 (18.68−24.16) | 20.78 (19.08−22.57) | 22.16 (19.89−24.57) | 18.86 (16.34−21.58) | 29.47 (27.55−31.45) | 31.52 (28.95−34.18) | 26.60 (23.72−29.63) |
| Public | 9.66 (8.02−11.50) | 10.85 (8.62−13.43) | 7.95 (5.69−10.75) | 7.93 (6.44−9.64) | 8.65 (6.65−11.02) | 6.90 (4.80−9.56) | 12.76 (10.89−14.82) | 13.05 (10.61−15.81) | 12.34 (9.53−15.63) |
| Private | 42.41 (39.29−45.57) | 46.48 (42.32−50.68) | 36.80 (32.14−41.66) | 35.98 (32.98−39.08) | 38.38 (34.36−42.52) | 32.69 (28.18−37.44) | 49.23 (46.06−52.41) | 53.70 (49.50−57.86) | 43.10 (38.27−48.03) |
| *Late Adolescents (15−19 years)* |  |  |  |  |  |  |  |  |  |
| Underweight |  |  |  |  |  |  |  |  |  |
| Total | 17.01 (13.78−20.64) | 19.48 (15.21−24.35) | 12.78 (8.28−18.55) | 30.12 (26.08−34.41) | 31.82 (26.65−37.34) | 27.22 (20.87−34.34) | 5.53 (3.68−7.95) | 5.84 (3.50−9.08) | 5.00 (2.31−9.28) |
| Public | 23.10 (18.48−28.26) | 25.77 (19.77−32.53) | 18.35 (11.58−26.91) | 39.93 (34.38−45.69) | 41.75 (34.73−49.03) | 36.70 (27.67−46.47) | 8.25 (5.41−11.94) | 8.76 (5.19−13.66) | 7.34 (3.22−13.95) |
| Private | 7.03 (3.79−11.72) | 8.77 (4.29−15.54) | 4.23 (0.88−11.86) | 14.05 (9.39−19.91) | 14.91 (8.93−22.80) | 12.68 (5.96−22.70) | 1.08 (0.13−3.85) | 0.88 (0.02−4.79) | 1.41 (0.04−7.60) |
| Overweight |  |  |  |  |  |  |  |  |  |
| Total | 8.81 (6.45−11.69) | 9.09 (6.13−12.87) | 8.33 (4.74−13.37) | 9.22 (6.81−12.14) | 10.06 (6.94−13.98) | 7.78 (4.32−12.71) | 14.96 (11.91−18.44) | 12.66 (9.16−16.90) | 18.89 (13.45−25.38) |
| Public | 5.28 (3.05−8.43) | 5.15 (2.50−9.28) | 5.50 (2.05−11.60) | 4.62 (2.55−7.63) | 4.64 (2.14−8.62) | 4.59 (1.51−10.38) | 7.92 (5.14−11.56) | 6.70 (3.62−11.19) | 10.09 (5.15−17.34) |
| Private | 14.59 (9.84−20.52) | 15.79 (9.63−23.80) | 12.68 (5.96−22.70) | 16.76 (11.68−22.93) | 19.30 (12.51−27.75) | 12.68 (5.96−22.70) | 26.49 (20.28−33.46) | 22.81 (15.47−31.60) | 32.39 (21.76−44.55) |
| Obesity |  |  |  |  |  |  |  |  |  |
| Total | 4.30 (2.68−6.50) | 4.87 (2.75−7.91) | 3.33 (1.23−7.11) | 3.28 (1.89−5.27) | 3.57 (1.80−6.30) | 2.78 (0.91−6.36) | 7.79 (5.57−10.53) | 9.42 (6.40−13.24) | 5.00 (2.31−9.28) |
| Public | 2.31 (0.93−4.70) | 2.06 (0.56−5.19) | 2.75 (0.57−7.83) | 2.31 (0.93−4.70) | 2.06 (0.56−5.19) | 2.75 (0.57−7.83) | 4.62 (2.55−7.63) | 5.15 (2.50−9.28) | 3.67 (1.01−9.13) |
| Private | 7.57 (4.20−12.37) | 9.65 (4.92−16.61) | 4.23 (0.88−11.86) | 4.86 (2.25−9.03) | 6.14 (2.50−12.24) | 2.82 (0.34−9.81) | 12.97 (8.49−18.69) | 16.67 (10.34−24.80) | 7.04 (2.33−15.67) |
| Overweight/Obesity |  |  |  |  |  |  |  |  |  |
| Total | 13.11 (10.25−16.44) | 13.96 (10.29−18.34) | 11.67 (7.37−17.28) | 12.50 (9.70−15.77) | 13.64 (10.01−17.98) | 10.56 (6.48−15.99) | 22.75 (19.10−26.73) | 22.08 (17.57−27.13) | 23.89 (17.86−30.80) |
| Public | 7.59 (4.87−11.17) | 7.22 (4.00−11.81) | 8.26 (3.85−15.10) | 6.93 (4.34−10.40) | 6.70 (3.62−11.19) | 7.34 (3.22−13.95) | 12.54 (9.03−16.81) | 11.86 (7.67−17.26) | 13.76 (7.91−21.68) |
| Private | 22.16 (16.40−28.84) | 25.44 (17.75−34.45) | 16.90 (9.05−27.66) | 21.62 (15.92−28.26) | 25.44 (17.75−34.45) | 15.49 (8.00−26.03) | 39.46 (32.37−46.90) | 39.47 (30.45−49.06) | 39.44 (28.03−51.75) |
| *Overall (6−19 years)* |  |  |  |  |  |  |  |  |  |
| Underweight |  |  |  |  |  |  |  |  |  |
| Total | 12.24 (11.22−13.32) | 13.86 (12.45−15.37) | 10.05 (8.64−11.61) | 26.04 (24.66−27.45) | 26.82 (24.98−28.71) | 24.98 (22.91−27.15) | 4.95 (4.29−5.69) | 4.92 (4.06−5.91) | 5.00 (3.99−6.16) |
| Public | 19.38 (17.65−21.20) | 21.53 (19.19−24.02) | 16.34 (13.86−19.06) | 38.50 (36.35−40.70) | 39.58 (36.75−42.47) | 36.98 (33.65−40.40) | 8.09 (6.92−9.38) | 8.07 (6.57−9.80) | 8.11 (6.33−10.20) |
| Private | 4.81 (3.89−5.88) | 5.55 (4.25−7.10) | 3.87 (2.66−5.42) | 13.07 (11.58−14.67) | 12.98 (11.02−15.15) | 13.18 (10.95−15.68) | 1.69 (1.16−2.38) | 1.51 (0.86−2.43) | 1.93 (1.11−3.12) |
| Overweight |  |  |  |  |  |  |  |  |  |
| Total | 13.93 (12.85−15.06) | 13.77 (12.36−15.28) | 14.14 (12.49−15.92) | 14.26 (13.17−15.41) | 14.09 (12.66−15.60) | 14.50 (12.83−16.30) | 15.66 (14.53−16.85) | 14.99 (13.53−16.54) | 16.58 (14.81−18.46) |
| Public | 5.65 (4.67−6.76) | 5.82 (4.54−7.33) | 5.41 (3.95−7.19) | 5.39 (4.44−6.48) | 5.47 (4.23−6.94) | 5.28 (3.85−7.05) | 7.63 (6.49−8.89) | 7.03 (5.62−8.66) | 8.48 (6.66−10.61) |
| Private | 22.53 (20.67−24.49) | 22.39 (19.92−25.02) | 22.73 (19.92−25.74) | 23.49 (21.60−25.47) | 23.42 (20.91−26.09) | 23.58 (20.72−26.62) | 24.02 (22.11−26.01) | 23.61 (21.09−26.28) | 24.55 (21.65−27.63) |
| Obesity |  |  |  |  |  |  |  |  |  |
| Total | 9.44 (8.54−10.41) | 11.15 (9.87−12.54) | 7.13 (5.93−8.48) | 5.71 (4.99−6.48) | 6.50 (5.51−7.61) | 4.63 (3.67−5.76) | 13.41 (12.35−14.52) | 15.76 (14.26−17.34) | 10.24 (8.81−11.81) |
| Public | 2.85 (2.16−3.68) | 3.30 (2.34−4.50) | 2.21 (1.32−3.47) | 1.78 (1.24−2.47) | 2.08 (1.34−3.08) | 1.35 (0.68−2.41) | 4.48 (3.61−5.49) | 5.38 (4.15−6.85) | 3.19 (2.10−4.65) |
| Private | 16.30 (14.66−18.04) | 19.66 (17.31−22.18) | 11.97 (9.84−14.38) | 9.79 (8.49−11.22) | 11.29 (9.45−13.35) | 7.86 (6.12−9.91) | 22.70 (20.83−24.65) | 27.00 (24.35−29.78) | 17.17 (14.66−19.92) |
| Overweight/Obesity |  |  |  |  |  |  |  |  |  |
| Total | 23.37 (22.04−24.73) | 24.92 (23.13−26.78) | 21.27 (19.31−23.33) | 19.97 (18.72−21.27) | 20.59 (18.92−22.33) | 19.13 (17.26−21.12) | 29.07 (27.64−30.53) | 30.74 (28.83−32.71) | 26.81 (24.68−29.03) |
| Public | 8.49 (7.30−9.82) | 9.11 (7.52−10.93) | 7.62 (5.89−9.66) | 7.17 (6.07−8.40) | 7.55 (6.09−9.23) | 6.63 (5.02−8.57) | 12.11 (10.70−13.63) | 12.41 (10.56−14.46) | 11.67 (9.55−14.08) |
| Private | 38.84 (36.63−41.08) | 42.05 (39.06−45.08) | 34.70 (31.46−38.06) | 33.28 (31.16−35.46) | 34.71 (31.85−37.66) | 31.44 (28.29−34.73) | 46.72 (44.45−49.00) | 50.61 (47.56−53.66) | 41.72 (38.33−45.16) |

*^#^ The World Health Organization, ^@^* *The International Obesity Task Force, ^$^* *The Indian Academy of Pediatrics*

**S3 Table:** Prevalence of underweight, overweight, obesity and overweight/obesity in different age groups by gender and school type using various criteria in children and adolescents aged 6-19 years (n=3856).

**S1 file: Operational definitions**

Underweight, overweight and obesity: The criteria used in children and adolescents were based on their BMI-for-age, using the World Health Organization (WHO) Growth reference 5-19 years [1], the International Obesity task force (IOTF) criteria [2–4] and the Indian Academy of Pediatrics (IAP) guidelines [5]. The IAP criteria do not provide a specific cut-off for underweight; therefore, the authors defined it as a BMI-for-age below the 5^th^ percentile, based on age and sex.

Central obesity: Waist circumference (cm) above 90^th^ percentile based on age and sex [6,7].

Childhood hypertension: Pre-hypertension was defined as SBP/DBP (mmHg) between 90^th^ to <95^th^ percentile and hypertension was defined as SBP/DBP (mmHg) at or above 95^th^ percentile based on age, sex and height [8].

Lipid abnormality: It was categorized based on the following parameters, TC (mg/dl), TG (mg/dl), low-density lipoprotein (LDL-c in mg/dl) and HDL-c (mg/dl): Total Cholesterol: normal if TC<170 mg/dl, borderline if TC between 170 mg/dl and 199 mg/dl and high if TC≥200 mg/dl; Triglyceride: normal if TG<90 mg/dl, borderline if TG between 90 mg/dl and 129 mg/dl and high if TG≥130 mg/dl; Low-density lipoprotein: normal if LDL-c<110 mg/dl, borderline if LDL-c between 110 mg/dl and 129 mg/dl and high if LDL-c≥130 mg/dl; and High-density lipoprotein: normal if HDL-c>45 mg/dl, borderline if HDL-c between 40 mg/dl and 45 mg/dl and low if HDL-c<40 mg/dl [9].

Impaired Fasting plasma glucose: It was defined as a FPG level>100 mg/dl [10].

Fasting insulin: High fasting insulin (hyperinsulinemia) was defined as a fasting insulin≥25 μIU/ml [11,12].

MetS: It was calculated using the National Cholesterol Education Program Adult Treatment Panel III (NCEP-ATP-III) and IDF criteria [6,13].

Dyslipidemia: It was defined as the presence of any lipid abnormality (high TC, TG, LDL-c and low HDL-c) [9].

Metabolically Obese Normal Weight (MONW) adolescents were defined as those who had a normal BMI-for-age but exhibited at least one metabolically abnormal trait used to define MetS. These traits include high triglycerides (TG≥110 mg/dl), low high-density lipoprotein cholesterol (HDL-c≤40 mg/dl), high fasting plasma glucose levels (FPG≥110 mg/dl), hypertension (SBP≥90^th^ percentile or DBP≥90^th^ percentile) or waist circumference (WC≥90^th^ percentile) [13–15]. Metabolically obese underweight (MOUW) adolescents were defined as those who had their BMI-for-age<5^th^ percentile according to the IAP criteria but exhibited at least one metabolically abnormal trait mentioned above.

References

1. de Onis M, Onyango AW, Borghi E, Siyam A, Nishida C, Siekmann J. Development of a WHO growth reference for school-aged children and adolescents. Bull World Health Organ. 2007;85: 660–667. doi:10.2471/blt.07.043497

2. Cole TJ, Bellizzi MC, Flegal KM, Dietz WH. Establishing a standard definition for child overweight and obesity worldwide: international survey. BMJ. 2000;320: 1240–1243. doi:10.1136/bmj.320.7244.1240

3. Cole TJ, Flegal KM, Nicholls D, Jackson AA. Body mass index cut offs to define thinness in children and adolescents: international survey. BMJ. 2007;335: 194. doi:10.1136/bmj.39238.399444.55

4. Cole TJ, Lobstein T. Extended international (IOTF) body mass index cut-offs for thinness, overweight and obesity. Pediatr Obes. 2012;7: 284–294. doi:10.1111/j.2047-6310.2012.00064.x

5. Khadilkar V, Yadav S, Agrawal KK, Tamboli S, Banerjee M, Cherian A, et al. Revised IAP growth charts for height, weight and body mass index for 5- to 18-year-old Indian children. Indian Pediatr. 2015;52: 47–55. doi:10.1007/s13312-015-0566-5

6. Zimmet P, Alberti KGM, Kaufman F, Tajima N, Silink M, Arslanian S, et al. The metabolic syndrome in children and adolescents ? an IDF consensus report. Pediatr Diabetes. 2007;8: 299–306. doi:10.1111/j.1399-5448.2007.00271.x

7. Xi B, Zong X, Kelishadi R, Litwin M, Hong YM, Poh BK, et al. International Waist Circumference Percentile Cutoffs for Central Obesity in Children and Adolescents Aged 6 to 18 Years. The Journal of Clinical Endocrinology & Metabolism. 2020;105: e1569–e1583. doi:10.1210/clinem/dgz195

8. Flynn JT, Kaelber DC, Baker-Smith CM, Blowey D, Carroll AE, Daniels SR, et al. Clinical Practice Guideline for Screening and Management of High Blood Pressure in Children and Adolescents. Pediatrics. 2017;140: e20171904. doi:10.1542/peds.2017-1904

9. Expert Panel on Integrated Guidelines for Cardiovascular Health and Risk Reduction in Children and Adolescents. Expert Panel on Integrated Guidelines for Cardiovascular Health and Risk Reduction in Children and Adolescents: Summary Report. Pediatrics. 2011;128: S213–S256. doi:10.1542/peds.2009-2107C

10. American Diabetes Association. Standards of Medical Care in Diabetes—2014. Diabetes Care. 2013;37: S14–S80. doi:10.2337/dc14-S014

11. Saxena P, Prakash A, Nigam A. Efficacy of 2-hour post glucose insulin levels in predicting insulin resistance in polycystic ovarian syndrome with infertility. Journal of Human Reproductive Sciences. 2011;4: 20. doi:10.4103/0974-1208.82355

12. Sullivan CS, Beste J, Cummings DM, Hester VH, Holbrook T, Kolasa KM, et al. Prevalence of hyperinsulinemia and clinical correlates in overweight children referred for lifestyle intervention. Journal of the American Dietetic Association. 2004;104: 433–436. doi:10.1016/j.jada.2003.12.020

13. Cook S, Weitzman M, Auinger P, Nguyen M, Dietz WH. Prevalence of a metabolic syndrome phenotype in adolescents: findings from the third National Health and Nutrition Examination Survey, 1988-1994. Arch Pediatr Adolesc Med. 2003;157: 821–827. doi:10.1001/archpedi.157.8.821

14. Ruderman NB, Schneider SH, Berchtold P. The “metabolically-obese,” normal-weight individual. The American Journal of Clinical Nutrition. 1981;34: 1617–1621. doi:10.1093/ajcn/34.8.1617

15. Green AK, Jacques PF, Rogers G, Fox CS, Meigs JB, McKeown NM. Sugar-sweetened beverages and prevalence of the metabolically abnormal phenotype in the Framingham Heart Study. Obesity (Silver Spring). 2014;22: E157-163. doi:10.1002/oby.20724

**S2 file: Model building strategy and commands used for analysis**

*** The prevalence of cardio-metabolic traits and its 95% CI were estimated using Stata 18.0 (StataCorp LP, USA) and the command used was:

. prop cmt^@^, citype(exact)

Strategy to build the model: The characteristics such as children’s age and gender, and father’s and mother education were compared between the private and public schools as these variables are known to have theoretical relevance and potential confounding effects. The distribution of these variables was statistically significant between the two school types. Therefore, we adjusted these variables in the random-effects logistic regression model to estimate the prevalence ratio of socioeconomic differential for each cardio-metabolic trait. We also assessed for the interaction effects (e.g., school type × gender, school type × parental education) but none were found to be statistically significant.

*** We used the following commands to explore the interaction effect of socioeconomic differential (school type) with children’s age and gender, and father’s and mother education on the cardio-metabolic traits using Stata 18.0 (StataCorp LP, USA).

. cc cmt^@^ sed^$^, by(em^#^)

The R commands used to build the random effect logistic regression model using R 4.3.3 (R Foundation of Statistical Computing, Vienna, Austria) are as follows:

### For overall analysis

> prLogisticDelta(cmt^@^~sed^$^+em^#^+(1|cv ^*^), data=data, pattern="marginal", cluster = TRUE)

^@^Cardio-metabolic traits- general obesity, central obesity, hypertension, dyslipidemia, impaired fasting plasma glucose, hyperinsulinemia, metabolic syndrome (NCEP ATP-III), metabolic syndrome (IDF), metabolically obese normal weight.

^$^Socioeconomic differential such as public and private schools.

^#^Effect modifier such as age, sex, father education and mother education.

^*^Cluster variable -school.

**Data Availability Statement:** The data used in this study are an extract from an ongoing longitudinal study and cannot be shared publicly before completion of the study. The data that support the findings of this study are available with the corresponding author and will be shared upon reasonable request to the Institute Ethics Committee (ethicscommitteeaiims@gmail.com) after completion of the study.
