# Supplementary material for: Cardio-metabolic traits and its socioeconomic differentials among school children including metabolically obese normal weight phenotypes in India: A post-COVID baseline characteristics of LEAP-C cohort
Source: PLoS One. 2025 May 6;20(5):e0321898. doi: 10.1371/journal.pone.0321898 (PMC12054912; doi:10.1371/journal.pone.0321898)
Supplement: Attachment — Submitted filename: Response to Reviewers.docx [file pone.0321898.s003.docx]

To

The Academic Editor

PLOS One

We thank the reviewers for their valuable comments that helped us to improve our manuscript. The following are the point-wise response to reviewers and the line numbers mentioned in the responses are as per the file labeled as ‘Manuscript’.

**Response to the Academic Editor**

When submitting your revision, we need you to address these additional requirements.

1. Please ensure that your manuscript meets PLOS ONE's style requirements, including those for file naming. The PLOS ONE style templates can be found at

<https://journals.plos.org/plosone/s/file?id=wjVg/PLOSOne_formatting_sample_main_body.pdf> and

[https://journals.plos.org/plosone/s/file?id=ba62/PLOSOne_formatting_sample_title_authors_affiliations.pdf](https://track.editorialmanager.com/CL0/https:%2F%2Fjournals.plos.org%2Fplosone%2Fs%2Ffile%3Fid=ba62%2FPLOSOne_formatting_sample_title_authors_affiliations.pdf/1/010f0194a5aa08b3-a3480d7f-4605-4e7c-a616-40ee4dfce8f6-000000/avey5ka9ssE-4SZWVeTJ1T_rLcIV6vczk3p5xkkdovc=195)

**Response: We revised the manuscript as per PLOS ONE's style requirements.**

2. We note that the grant information you provided in the ‘Funding Information’ and ‘Financial Disclosure’ sections do not match.

When you resubmit, please ensure that you provide the correct grant numbers for the awards you received for your study in the ‘Funding Information’ section.

**Response: We corrected this also.**

3. Thank you for stating the following financial disclosure:

 “Indian Council of Medical Research, Department of Health Research, Ministry of Health and Family Welfare, Government of India, funded the conduct of the study”

Please state what role the funders took in the study.  If the funders had no role, please state: "The funders had no role in study design, data collection and analysis, decision to publish, or preparation of the manuscript."

If this statement is not correct you must amend it as needed.

Please include this amended Role of Funder statement in your cover letter; we will change the online submission form on your behalf.

**Response: As suggested, we have included the Role of Funder statement in the cover letter.**

4. Thank you for stating the following in the Acknowledgments Section of your manuscript:

“This study is funded by the Indian Council Medical Research, Department of Health Research, Ministry of Health and Family Welfare, Government of India. We gratefully acknowledge the Director (Education), New Delhi Municipal Council, school principals and teachers for granting the permission and their support for the conduct of the study. We wish to thank all the field data collection and laboratory investigation teams. We wish to gratefully acknowledge Senthil Amudhan, National Institute of Mental Health Sciences, Bengaluru, India. We also wish to thank all the study participants for their support and cooperation for completing this study. We also wish also thank the REDCap administrators for providing user account and its management for storing the study data. “

We note that you have provided funding information that is not currently declared in your Funding Statement. However, funding information should not appear in the Acknowledgments section or other areas of your manuscript. We will only publish funding information present in the Funding Statement section of the online submission form.

Please remove any funding-related text from the manuscript and let us know how you would like to update your Funding Statement. Currently, your Funding Statement reads as follows:

  “Indian Council of Medical Research, Department of Health Research, Ministry of Health and Family Welfare, Government of India, funded the conduct of the study”

Please include your amended statements within your cover letter; we will change the online submission form on your behalf.

**Response: We removed the funding related information from the manuscript.**

5. In the online submission form, you indicated that [Data cannot be shared publicly because data contains confidential participant information. Data are available on request from the corresponding author.].

All PLOS journals now require all data underlying the findings described in their manuscript to be freely available to other researchers, either 1. In a public repository, 2. Within the manuscript itself, or 3. Uploaded as supplementary information.

This policy applies to all data except where public deposition would breach compliance with the protocol approved by your research ethics board. If your data cannot be made publicly available for ethical or legal reasons (e.g., public availability would compromise patient privacy), please explain your reasons on resubmission and your exemption request will be escalated for approval.

**Response: We included the revised statement in the cover letter and updated it in the online submission form also.**

6. Please review your reference list to ensure that it is complete and correct. If you have cited papers that have been retracted, please include the rationale for doing so in the manuscript text, or remove these references and replace them with relevant current references. Any changes to the reference list should be mentioned in the rebuttal letter that accompanies your revised manuscript. If you need to cite a retracted article, indicate the article’s retracted status in the References list and also include a citation and full reference for the retraction notice.

**Response: We checked the Reference list and edited as necessary. We thank the Academic Editor for all the insights.**

**Review Comments to the Author:**

**Reviewer #1:** The manuscript is written in a lucid manner. The authors have addressed the need/importance in a clear elaborate manner. The operational definitions could be attached as an appendix. Sample size could be mentioned within the parentheses () for all table headings. The manuscript could be "ACCEPTED" for publication.

**Response: We thank the reviewer for the encouraging comments and evaluating our manuscript. The sample size has been mentioned in the heading for Table 1 and for Tables 2-5, it is given inside the tables as it is different for physiological, clinical and biochemical parameters.**

**Reviewer #2:** The study describes a technically sound piece of scientific research with data that supports the conclusions. The work is original and addresses an important public health issue in the context of dual burden of disease that the low- and middle-income countries are currently faced with. Comparing two socio-economic groups is a valuable insight, particularly the use of public Vs private as basis for categorizing socio-economic status looks an interesting venture. The methods are well explained and appear replicable. The sample sizes are adequate. The conclusions are drawn appropriately based on the data presented.

The quality of the manuscript could improve substantially if the authors can address some of the issues raised below.
**We thank the reviewer for evaluating our manuscript vividly and helping us to improve the manuscript.**
Abstract
**Comment 1:** In the second sentence of the abstract, the authors state that; “Further, these risks may have aggravated due to worsening food security and diet quality during the pandemic”. The starting word, “Further” could be removed as it adds no value to the meaning of the sentence yet occupies space. Please be specific by ending this statement using ....... during the COVID-19 pandemic.

**Response: We thank the reviewer for his suggestion. Lines 26-27 has been edited.**

**Comment 2:** The statement: “Among 3888 (77.22%) recruited, 1985 (93.02%) were from public schools and 1903 (66.00%) were from private-schools aged 6-19 years” is unclear. What was the denominator, given that 77.22% and 66.00% appear not to have a common denominator? Please revise that sentence and make it clear to the reader.

**Response: Lines 41-42 has been revised and edited as suggested.**

**Comment 3:** The authors further state that; “The prevalence of underweight and general obesity was 4.95% (95% CI 1.25-12.72) and 13.41 (95% CI 2.98-33.87) respectively with higher underweight prevalence in public schools (p<0.0001) and obesity prevalence in private-schools (p<0.0001) with adjusted PR=4.42, 95% CI 2.90-6.72)”. Given that the statement refers to prevalence of 2 variables, the word was may not be most suitable. Please replace it with “were”. As differences in prevalence of underweight and obesity were statistically significant, it will be good for the authors to say; ........ respectively with significantly higher underweight prevalence in public schools (p<0.0001) and obesity prevalence in private-schools (p<0.0001) with adjusted PR=4.42, 95% CI 2.90-6.72)”. Please, also be clear what the PR at the end of this sentence represents given the mix in the sentence. The authors might need to consider breaking this long sentence into shorter sentences for clarity.

**Response: We thank the reviewer for bringing the clarity in the abstract results. Lines 42-48 has been revised.**

**Comment 4:** The sentence; “The association with type of schools was also significant for impaired fasting plasma glucose (adjusted PR=2.37, 95% CI: 1.19-4.72) and metabolic syndrome (adjusted PR=3.51, 95% CI 1.54-8.01)” is not clear. Please revise and make this clear. Please state the type of association in reference and the direction with reference to the independent variable (in this case the type of school).

**Response: Lines 50-52 has been revised as suggested.**

**Comment 5:** The authors conclude that; “An effective implementation of food security measures and targeted initiatives will be crucial to mitigate the socio-economic and gender disparities associated with growing burden of cardiometabolic traits”. The reference to gender in this conclusion is not substantiated by results. The authors should consider including some gender differences in the results or remove the reference to gender in this conclusion.

**Response: We agree with the reviewer in this context and Line 64 has been edited as suggested.**

Data Availability:

**Comment 6:** Regarding data availability, the authors state that some restriction may apply regarding data availability. It is important the authors provide a justification as to why data availability is limited. It is now a good research practice to make research data available to readers and other researchers. The authors add that: “Data cannot be shared publicly because data contains confidential participant information. Data are available on request from the corresponding author”. The authors should consider anonymizing the data before sharing it unless there are unavoidable impediments to anonymizing the data or sharing it.

**Response: We thank the reviewer for his valuable input and we agree that making data available to readers and researchers is immensely important and a valuable practice. In this manuscript, we have provided all summary data using baseline data of the ongoing longitudinal study either in the manuscript itself or as supplementary information. Since the study is still in progress, the disclosure of raw data will be subject to the policies of the funding agency and the authors' institution.**

INTRODUCTION
**Comment 7:** The introduction is generally well written, apart from a few grammatical errors. The authors should consider a review and revision of grammar by someone whose first language is English for both the abstract and introduction. Adding information on the prevalence of under- and overweight in low- and middle-income countries would give perspective to the background.

**Response: We appreciate and value the comments of the reviewer. We have added the prevalences of underweight, overweight and obesity in low-and middle-income countries (Lines 75-77) in the introduction. We also revised the Abstract and Introduction as suggested.**

METHODS:
**Comment 8:** It is particularly concerning that the study schools were conveniently sampled and this has an implication on the generalizability of the findings. The authors should consider acknowledging this as a limitation of the study, although the discussion states otherwise.

**Response: As suggested, we have added this as a limitation and revised statement in the discussion (Lines 475-476).**

**Comment 9:** The “Study setting and recruitment strategy” does not have information on setting. The study setting would for example, explain the total number of schools, number private, then public etc. Such information is missing under this section. The authors should consider including such context or remove ‘study setting’ from the sub-section header. The ‘was’ in line 157 should be were.

**Response: We thank the reviewer for his suggestion. Lines 141-145 and 156 has been revised and edited.**

**Comment 10:** Lines 165 and 166 refer to ethical clearance/study approval under the inclusion and exclusion criteria. Please create a separate sub-section for the ethical consideration and clearance/approval. The authors have not explained what was done regarding those who did not consent or those discontinued. Were these considered as non-response or replaced? What of those who withdrew after start of the interview/measurements? Please explain what the study did under such circumstances.

**Response: We thank the reviewer for his valuable comment. Ethical clearance/study approval has been added as a separate sub-section titled, ‘Ethical consideration and approval’ in Lines 165-174.** **Lines 175-181 provide the criteria for non-respondents and those who discontinued. Out of 5045 eligible students, 3888 (77.07%) participated in the survey (private =1903 and public=1985). The non-response rate in private and public schools were 34.45% and 6.98% respectively. These non-respondents include students who did not get consent, consented but absent and did not give assent (after start of the interview).** **To increase the response rate, multiple reminders were sent to those students’ parents by email/WhatsApp class group ids and a study representative conducted sessions with the parents during parents-teacher meeting also. Non-response was higher among private school students than public school students, primarily due to parental hesitance about medical procedures like blood sampling in a school setting. This reluctance, particularly among parents from higher socioeconomic backgrounds, was heightened by the unprecedented circumstances of the COVID-19 pandemic. We added this in the discussion in Lines 476-482.**

**Comment 11:** Lines 261 and 262: The authors state; “Finally, the total sample size for anthropometry parameters was1980 and for biochemical parameters is 1100 only from public and private schools”. It is not clear if these (1980 and 1100) were independent samples. If so, why didn’t the authors opportunistically consider 1980, the higher of the two as the sample size to cater for both groups?

**Response: The focus of the study for the blood pressure/biochemical parameters were to include a subset of students aged 11-15 years from the total sample size of 1980. This selection was made because the focus of the study was only among 11–15-year age group for these parameters. It has now been clarified/corrected in the manuscript; please refer to Lines 245-248.**

**Comment 12:** Ethical consideration: What happened to those detected as underweight, overweight, obese, hypertensive, with low HDL or high LDL etc? Did the study consider managing, referring or providing appropriate counselling as part of ethical responsibilities? It is important that a sub-section is created in the methods section, detailing how such ethical issues were handled, alongside the study approval, that I earlier requested to be separated from inclusion and exclusion criteria.

**Response: We appreciate the reviewer’s insightful suggestion. Individualized measurement reports, sealed in envelopes, were distributed to all participants through their schools. The list of participants with abnormal findings was provided to the respective school principals, who were asked to inform parents during the parent-teacher meeting. During this meeting, we requested the teachers to highlight the importance of physical activity, a balanced diet, and reduced screen time to support the children's well-being. The team had no direct role in counselling, as the school authorities did not permit them to interact with the participants' parents. However, the team offered medical support to parents who requested assistance for their children. Lines 165-174 has been revised and edited accordingly.**

**Comment 13:** The authors express using random effects logistic regression models. However, the manuscript does not discuss the model building strategy, and how well the study data fitted the model. Such description would provide confidence in the results provided. Alternatively, the model building strategy and fitting tests can be submitted as supplementary material. Plus, some independent variables have abruptly surfaced in the results, when they were not discussed in the methods. It is important that each dependent variable, and the corresponding independent variables are discussed in the methods so that the reader knows what to expect in the results.

**Response: We thank the reviewer for bringing clarity in the methods. The random effects logistic regression with marginal standardization was done to assess the association between cardio-metabolic traits and socioeconomic differential (school types) adjusting for some of the known confounders such as children’s age, sex and their parent’s education. Detailed information on each dependent and independent variable used in this model is provided in Lines 266-270.**

RESULTS
**Comment 14:** The 95% CI for prevalence of obesity (lines 331-335) are unusually too wide, despite prevalence estimates away from 50%. The authors need to account for this as it puts the precision of estimates to question. The authors should consider merging categories if this (wide 95% CI) is associated with smaller sample sizes across sub-groups. In addition, there appears to be differences between the lower and upper limits of the 95% CIs, with lower values having smaller difference from the estimated prevalence. The authors should provide explanation(s) for such findings.

**Response: The 95% CI for prevalence of obesity are unusually too wide as the standard error was corrected accounting for the complex survey design with clustering. Additionally, when the prevalence is low or high, not around 50%, the distribution of the estimated prevalence can become skewed causing asymmetric CI. The lower limit may be closer to the estimate (prevalence cannot go below 0%) but upper limit has more room to expand leading to asymmetric CI. Now, we have reanalysed the data to calculate the standard error without accounting for clustering and calculated 95% CI using exact method. We have corrected the Lines 261-262 in the ‘Data management and statistical analysis’ sub-section and ‘Result’ section and Tables 2-5 wherever required. We appreciate and thank the reviewer for this insightful comment.**

**Comment 15:** The concept of early and late adolescents was not explained anywhere in the methods, and suddenly surfaces in the results. Please explain (either in the methods or where those results are presented) the age categories representing these (early and late) sub-groups of adolescents.

**Response: The explanation for sub-groups of adolescents has been provided in ‘Data management and statistical analysis’ sub-section in Line 259-260 as suggested by the reviewer.**

**Comment 16:** Table 2 has ‘Fating’ plasma glucose (mg/dl) instead of fasting .... Please check the different tables and correct this across all.

**Response: We thank the reviewer for spotting the typo. All tables and figures have been checked and necessary correction has been done.**

**Comment 17:** In Table 2, NCEP ATP-III has unusually very high 95% CI among private school respondents. The authors need to explanation such an anomaly. Plus, it is interesting that some variables have overlap in 95% CI, while the p-values depict significant differences (i.e. p<0.05). The authors should consider explaining this.

**Response: The 95% confidence interval (CI) provided here corresponds to the estimate, i.e., prevalence, while the p-value tests the difference between groups (public and private schools). For instance, the individual CIs for public and private schools may overlap, but the CI for the difference between them may exclude zero, indicating statistical significance. When CIs overlap, statistical significance depends on the specific test used and the sample size. In cases where CIs do not overlap, statistical significance is almost always assured, though the sample size and test choice still influence the exact p-value.**

**Comment 18:** Lines 394-395: The authors state that; “The difference in prevalences of dyslipidemia was significantly different among girls (34.90% vs 26.61%, p=0.019) (Table 4)” without specifying the comparison groups. Please make it clear that this is between public and private schools. Use of the description ‘significantly different among girls’ is not clear. Please make it explicit that dyslipidemia prevalence is significantly higher in public than private schools or between girls and boys.

**Response: Lines 394-397 has been revised and edited.**

**Comment 19:** Lines 398-400: “The prevalence of MetS among urban adolescents according to ATP-III criteria and IDF criteria was 3.43% (95% CI 1.04-8.14) and 1.39% (95% CI 0.17-4.87) respectively”. Please specify the gender/sex these specific results refer to.

**Comment 20:** Lines 400-402: The authors state: “It was significantly lower among public school students compared to private school students (p=0.020) with adjusted PR of 3.51 (95% CI 1.54-8.01) (Table 2 and Fig 3). A similar pattern (Tables 3 and 4) was observed among boys (p=0.022) and girls (0.088)”. Please revise these statements to reflect the gender the results they are referring to.

**Comment 21:** By stating; “A similar pattern (Tables 3 and 4) was observed among boys (p=0.022) and girls (0.088)”, it is also implied that the difference is statistically significant in both gender’s results. However, the p-value of 0.088 among girls states otherwise. Please revise this.

**Comment 22:** Regarding Mets, it is also important to present results based on the standards in reference. I am seeing a simultaneous and indiscriminate use of ATP-III criteria and IDF results, and this risk causing confusion in interpretation, let alone raising the sensitivity regarding identification of Mets. It is important to consider results of each standard independently.

**Response (Comments 19 – 22): We thank the reviewer for all his suggestions regarding the prevalence of MetS and bringing clarity. Lines 400-410 has been revised as suggested.**

DISCUSSION
**Comment 23:** Line 434: “To our knowledge, this is the first longitudinal school-based study comprehensively assessed the ..........” ‘That’ appears to be missing between study and comprehensively, making the sentence lose meaning.

**Response: Line 442 has been edited as suggested.**

**Comment 24:** Line 438: “The findings revealed that the anthropometric, blood pressure,.....”. Should be ‘anthropometric measurements’.

**Comment 25:** Lines 438-440: The authors state that; “The findings revealed that the anthropometric, blood pressure, lipid levels, fasting plasma glucose and insulin were significantly higher among private school going students (proxy for higher SES) compared to public school students (proxy for lower SES)”. Please review this summary and exclude those measures whose differences were not statistically significant. Plus, it is important to make it clear in the methods section the analysis you considered as final such that discussion will focus on them. As it currently stands, the authors appear to be picking any statistically significant findings across the different analyses. Multi-variate analysis (i.e. the random effects logistic regression) could take precedence as the possible co-founders are controlled for in that (high) level of analysis.

**Response (Comments 24-25): We thank the reviewer for their suggestions. We agree that the multivariate analysis takes precedence, as it accounts for potential confounders. Therefore, we have briefly summarized the findings in the discussion in line with the study objectives.**

**Comment 26:** Lines 440-442: The statement; “The prevalence of underweight (nearly five times) was higher in public schools while obesity was higher in private school (more than five times) students as compared to their counterparts” is not clear. Please revise and make it clear to the reader.

**Response: Lines 446-448 has been revised.**

**Comment 27:** Lines 461-462: The authors state that: “These findings will be crucial to understand the adverse impact of COVID-19 on nutrition particularly on the vulnerable and marginalized groups”. Although this study was conducted in the post-COVID era, there is no evidence linking the findings to the COVID-19 pandemic given a lack of baseline pre-pandemic. The authors may consider removing reference to the ‘adverse impacts of COVID-19’ in the discussion. Alternatively, the effect/impact of COVID-19 can be discussed in reference to life-style changes following the lock-downs, not necessarily attributing the observed findings to it as implied here.

**Response: Lines 468-470 has been revised as suggested.**

**Comment 28:** Lines 478-479: The authors state: “Similarly, the current study reported lower rates of underweight prevalence among private school children reported previously”. This statement should end with ......... than reported previously. ‘Than’ is missing. The statement between lines 504-507 ends with a coma (not full stop). Please use a full stop.

**Response: Both the Lines 494 and 522 has been corrected.**

**Comment 29:** Between lines 549 to 551, the authors say the LEAP study will make them establish a causal relationship by assessing determinants of clustering of CVD risks. Although this is a cohort study, it is worth taking note that ‘causal relationship studies’ take on some stringent criteria. The authors should consider revising their choice of the language, ‘causal’ as opposed to an association, unless they are sure the design will deliver a ‘causal relationship’.

**Response: We thank the reviewer for his valuable input. Lines 564-566 has been revised and edited.**

**Comment 30:** Lines 552-554: Please punctuate the sentences therein. Some full stops are being placed within continuing sentences.

**Response: We thank the reviewer for his suggestions. Line 569 has been edited.**

**GENERAL COMMENTS**

Charts/Figures: In their current state, the figures are not clearly visible. The authors should consider submitting high-resolution figures/charts.

**Response: We have uploaded all the figures to the PACE digital diagnostic tool and made necessary changes to ensure that figures meet PLOS requirements.**

The write-up contains several typos and grammatical errors. The authors should consider a review and revision by someone whose first language is English in order to improve the grammar and overall write-up.

**Response: As suggested, the necessary review and revision of the manuscript has been done to correct it.**
