# Supplementary material for: Cardio-metabolic traits and its socioeconomic differentials among school children including metabolically obese normal weight phenotypes in India: A post-COVID baseline characteristics of LEAP-C cohort
Source: PLoS One. 2025 May 6;20(5):e0321898. doi: 10.1371/journal.pone.0321898 (PMC12054912; doi:10.1371/journal.pone.0321898)
Supplement: Attachment — Submitted filename: Response to Reviewers..docx [file pone.0321898.s004.docx]

**Response to Reviewer**

We thank the reviewers for their valuable comments that helped us to improve our manuscript. The following are the point-wise response to reviewers and the line numbers mentioned in the responses are as per the file labeled as ‘Manuscript’.

**Review Comments to the Author**

**Reviewer #2:** The data for this study is not publicly available. This is understandable given that the data used in this study/analysis is an extract of data from an on-going study for which raw data may not be made public. It is good to note that the authors accept to make the study data available to individuals who request for them.

**Response: We agree with the reviewer. We are uploading the following for Data Availability:** **“The data used in this study is an extract from an ongoing study and is not currently publicly available. The data that support the findings of this study are available on reasonable request from the corresponding author subject to data sharing policy and ethical restrictions of the institute”.**

**Lines 140-141:** Under the study setting and recruitment strategy, the authors state that; “The study setting comprised five schools in Delhi, India, including three public and two private schools (convenient selection as schools were reluctant due to the pandemic).” Please note that this does not describe the ‘study setting’. I guess the five schools (three public and two private) is the sample size of schools. Study setting means the physical, social, and cultural context where the research is conducted. In the case of this study, the setting would (for example) mean describing the total number of schools in Delhi. Out of the total schools, what number are private and public. What is the proportion of the population going to private Vs public schools (if such data exists). Then it is also good context to add that in India (Delhi in this case), it is general practice that children from wealthy or higher socioeconomic groups generally go to private schools, while those from the poorer or lower socioeconomic groups go to public schools, citing appropriate sources where applicable. This would amount to setting.

Please distinguish your sample from study setting because the setting refers to the natural context (set-up) in the study area. As I stated in my initial review, if you cannot add the context, please consider removing ‘Study setting’ from the title “Study setting and recruitment strategy”. Recruitment strategy should suffice in this case. Please, also remove ‘setting’ from description of the sample as in line 140.

**Response: I thank the reviewer and completely agree with him. As suggested by the reviewer, we have removed ‘Study setting’ from the title “Study setting and recruitment strategy” and also “setting” from line 140.**
